# The Regulatory Role of Non-coding RNA in Autophagy in Myocardial Ischemia-Reperfusion Injury

**DOI:** 10.3389/fphar.2022.822669

**Published:** 2022-03-17

**Authors:** Dan Wang, Zhenchao Niu, Xiaolong Wang

**Affiliations:** ^1^ Cardiovascular Research Institute of Traditional Chinese Medicine, Shuguang Hospital Affiliated to Shanghai University of Traditional Chinese Medicine, Shanghai, China; ^2^ Cardiovascular Department of Traditional Chinese Medicine, Shuguang Hospital Affiliated to Shanghai University of Traditional Chinese Medicine, Shanghai, China; ^3^ Shuguang Hospital Affiliated to Shanghai University of Traditional Chinese Medicine, Branch of National Clinical Research Center for Chinese Medicine Cardiology, Shanghai, China

**Keywords:** non-coding RNA, autophagy, myocardial ischemia-reperfusion injury, mTOR, mitophagy

## Abstract

Following an acute myocardial infarction (AMI), thrombolysis, coronary artery bypass grafting and primary percutaneous coronary intervention (PPCI) are the best interventions to restore reperfusion and relieve the ischemic myocardium, however, the myocardial ischemia-reperfusion injury (MIRI) largely offsets the benefits of revascularization in patients. Studies have demonstrated that autophagy is one of the important mechanisms mediating the occurrence of the MIRI, while non-coding RNAs are the main regulatory factors of autophagy, which plays an important role in the autophagy-related mTOR signaling pathways and the process of autophagosome formation Therefore, non-coding RNAs may be used as novel clinical diagnostic markers and therapeutic targets in the diagnosis and treatment of the MIRI. In this review, we not only describe the effect of non-coding RNA regulation of autophagy on MIRI outcome, but also zero in on the regulation of non-coding RNA on autophagy-related mTOR signaling pathways and mitophagy. Besides, we focus on how non-coding RNAs affect the outcome of MIRI by regulating autophagy induction, formation and extension of autophagic vesicles, and the fusion of autophagosome and lysosome. In addition, we summarize all non-coding RNAs reported in MIRI that can be served as possible druggable targets, hoping to provide a new idea for the prediction and treatment of MIRI.

## Introduction

Acute myocardial infarction (AMI) is one of the cardiovascular conditions with a high mortality rate worldwide. The most effective therapeutic intervention is relieving coronary artery occlusion on time through thrombolysis, coronary artery bypass grafting or primary percutaneous coronary intervention (PPCI), which can restore the blood flow and promote myocardial perfusion (Amani et al., 2017). However, the cardiomyocyte damage is further aggravated after the reperfusion. This phenomenon is called myocardial ischemia-reperfusion injury (MIRI), which overturns the benefits of revascularization and increases the mortality and rehospitalization rates (Kulek et al., 2020).

It was found that lethal myocardial reperfusion injury accounted for nearly 50% of the final size of the acute myocardial infarction in the AMI animal models (Yellon and Hausenloy, 2007). Timely opening the infarcted arteries for revascularization can only reduce the infarct size of the myocardial area at risk (the hypo-perfused myocardial zone during myocardial infarction) by 50% (Lavandero et al., 2015). Thus, the prevention of the myocardial reperfusion injury will be critical in the treatment of AMI. Current therapies for MIRI include ischemic conditioning (e.g., ischemic preconditioning, hypothermia and remote ischemic preconditioning), physical conditioning (e.g., hypothermia), and medications (e.g., cyclosporin A and sodium nitrite, which improve mitochondrial function) (Hausenloy and Yellon, 2013; Davidson et al., 2019). However, their clinical therapeutic effects are not satisfactory. Further research exploring the mechanisms underlying MIRI development and new treatment strategies are needed.

At present, there is limited consensus on the pathogenesis of MIRI injury, and the reported mechanisms included reactive oxygen species (ROS) generation, Ca2+ overload, inflammatory response, microthrombosis, apoptosis, autophagy, etc.(Yang et al., 2019; Chang et al., 2021). Among them, autophagy is a natural defense and stress regulatory process that commonly occurs in the eukaryotic cells. This cellular process is evolutionarily conserved, which removes the misfolded or aggregated proteins and damaged or aging organelles formed under unfavorable conditions such as nutrient deficiency or energy crisis to maintain cell homeostasis (Lavandero et al., 2015). According to the different packaging cargos and transportation modes, autophagy can be classified into macroautophagy, microautophagy, chaperone-mediated autophagy, newly discovered DNA autophagy (Fujiwara et al., 2013b), and RNA autophagy (Fujiwara et al., 2013a). Usually, autophagy refers to macroautophagy.

A large amount of experimental evidence supports that autophagy is upregulated following MIRI, which occurs during myocardial ischemia and reperfusion. Autophagy was once been considered as a protective mechanism for cell homeostasis. An increasing number of studies have found that moderate autophagy occuring during the ischemic stage is beneficial to the cardiomyocytes while excessive autophagy during the reperfusion stage is harmful and even leads to cell death (Bainey and Armstrong, 2014). Accordingly, the degree of autophagy is critical to its protective or damaging effect during MIRI.

Non-coding RNAs (ncRNAs), once considered to be the “genomic junk”. However, accumulating pieces of evidence have been shown that non-coding RNAs are involved in the regulation of cell development, cell cycle, differentiation, apoptosis, and other critical cellular events. Among the ncRNAs, microRNAs (miRNAs) regulate a variety of cellular functions by promoting degradation or inhibiting the translation of target mRNAs (Condorelli et al., 2014). Long non-coding RNAs (lncRNAs) are involved in a wide range of biological processes such as chromatin remodeling, histone modification, DNA methylation, mRNA splicing and translation, as well as gene regulation at almost all levels (Yoon et al., 2014). Additionally, they can bind to the target mRNAs to promote or block their translation or act as an endogenous sponge of miRNAs to inhibit the negative regulation of miRNA on the expression of target genes (Ebert and Sharp, 2010). Recently discovered circular RNAs (circRNAs), which are rich in miRNA response elements, can also affect the expression of miRNA target genes as competitive endogenous RNA (ceRNA). The aforementioned non-coding RNAs have been shown to play critical roles in the MIRI, making them potential therapeutic targets. In this review, we aimed to explore how the non-coding RNAs affect the MIRI through the regulation of various phases of autophagy and to provide a new idea for prediction and treatment of MIRI.

### Regulation of mTOR Signaling Pathway by Non-coding RNA

The mammalian target of rapamycin (mTOR) is a protein kinase that regulates autophagy during myocardial ischemia. As a portal of the molecular regulator, it negatively regulates the process of autophagy at the initial stage (Laplante and Sabatini, 2012). Presently, there are mainly two mTOR-related signaling pathways, AMPK/mTOR and PI3K/AKT/mTOR signaling pathways. The mTOR signaling regulates the formation of the mTORC1 and mTORC2 complex and initiates autophagy in response to nutrition-, energy-, and stress-induced stimuli. The non-coding RNAs, a novel regulator of the mTOR pathway, regulate autophagy by targeting various molecules in the mTOR signaling pathway.

### PI3K/AKT/mTOR-Related Signaling Pathways

Phosphatidylinositol 3-kinase (PI3K) serves as a bridge, linking extracellular signals and cell responses and regulates cell growth, apoptosis, protein synthesis, cell metabolism, and other biological processes *via* controlling its downstream target kinase protein kinase B (AKT) (Hemmings and Restuccia, 2012).Growth factors such as insulin can activate the PI3K/AKT signaling pathway, and activated AKT directly phosphorylates and activates the mTOR molecule, thus inhibiting autophagy (Lum et al., 2005). It was found that the inhibition of miR-208a (Shi et al., 2020) and miR-30a (Li et al., 2016) inhibited the activation of mTOR molecules, which promoted the enhancement of mTOR-mediated autophagy, and protected the cardiomyocytes by inhibiting the PI3K/Akt signaling pathway. In addition, inhibition of miR-208a also increased the level of superoxide dismutase (SOD) and inhibited the expression of malondialdehyde (MAD), while miR-30a could also bind to the 3′-untranslated region (3′-UTR) of Beclin-1 mRNA, thus inhibiting Beclin-1 mediated autophagy (Wang et al., 2014). In contrast, the overexpression of miR-494 (Ning et al., 2020) and miR-384 (Zhang et al., 2019a) reduced the autophagy and apoptosis, enhanced the activity of cardiomyocytes, and protected the cardiomyocytes from I/R injury by activating the PI3K/AKT/mTOR signaling pathway. Besides, the regulation of the PI3K/AKT/mTOR signaling pathway by miR-494 is mediated by silencing information regulator 1 (SIRT1). The SIRT1 is the deacetylase of Atg7 protein, which is involved in the extension of the autophagosome membrane and promoting autophagy. It is speculated that miR-494 can modulate SIRT1, thus playing an important role in the autophagy extension stage.

Phosphatase and tensin homolog deleted on chromosome 10 (PTEN) is a lipid phosphatase that can dephosphorylate phosphatidylinositol (3,4,5)-trisphosphate (PIP3, PI3K product) and convert it into phosphatidylinositol (4,5)-bisphosphate (PIP2), which reduces Akt activation, exerting a negative regulatory effect on the PI3K/Akt/mTOR signaling pathway (Gericke et al., 2006). The exosomes extracted from the mouse bone marrow mesenchymal stem cells (BMMSCs) contain high concentrations of miR-29C. It has been found that miR-29C targets PTEN to activates the PTEN/AKT/mTOR pathway, thereby partially blocking the cardiac autophagy and protecting the myocardium from the ischemia/reperfusion (I/R) injury (Li et al., 2020a).

### AMPK/mTOR-Related Signaling Pathways

AMP-activated protein kinase (AMPK), another key molecule for sensing cellular nutritional status, has also been shown to be involved in the regulation of autophagy (Samari and Seglen, 1998). when cells are exposed to environmental stressors such as hypoxia, glucose deficiency and starvation during ischemia, intracellular energy decreases, causing decreased AMP/ATP ratio and AMPK activation (Hardie, 2015). Activated AMPK contributes to the phosphorylation of the Raptor subunit of the mTORC1 complex to inhibit mTORC1, indirect activating the ULK1 and inducing autophagy (Gwinn et al., 2008). It can also directly phosphorylate multiple serine sites in the central region of the ULK1 to activate ULK1 (Egan et al., 2011; Kim et al., 2011). Moreover, the AMPK can regulate autophagy through phosphorylation of the Atg13 (Puente et al., 2016).

The AMPK activity is regulated by phosphorylation of the Thr-172 (α-subunit) of the upstream liver kinase B1 (LKB1), and the mouse protein 25 (MO25) is one of the core members of the LKB1 complex, which can assist the LKB1 to exert its activity (Chen et al., 2012). MiR-429 was found to be prominently down-regulated both in the insulin-resistant mice and the hypoxia/reoxygenation (H/R)-treated cells. Inhibition of the miR-429 upregulated LKB1 and pAMPKα, enhancing autophagy. Further studies showed that miR-429 directly targeted MO25 to antagonize its activation on the MO25/LKB1/AMPK pathway, thereby enhancing autophagy, and ameliorating the H/R injury of the cardiomyocytes (Zhu and Hu, 2019).

However, another study reported that inhibition of the AMPK/mTOR/ULK1 pathway suppressed autophagy and reduced the MIRI in the H/R model of H9c2 cardiomyocytes, and this pathway was negatively regulated by miR-139-5p (Wang et al., 2017). In addition, Li et al. found that the AMPK/Nampt signaling pathway alleviated the I/R injury by inhibiting myocardial apoptosis and autophagy, and the reduced expression of the miR-206 was implicated in the activation of the AMPK/Nampt signaling pathway (Li et al., 2020b). Nampt has been proven to be an important regulator of autophagy, and its downregulation inhibited autophagy (Hsu et al., 2009). Li et al. found that the miR-300 activated the AMPK/mTOR signaling pathway by inhibiting Nampt and autophagy in sepsis (Li Y. et al., 2018). Thus, it is speculated that the role of the miR-206 in the AMPK/Nampt signaling pathway is also closely related to mTOR.

Matsui et al. (2007) found that autophagy induction during ischemia was AMPK-dependent. The AMPK-induced autophagy during ischemia provided a protective role by releasing free fatty acids and amino acids through degradation of the nonfunctional cytoplasmic proteins and generating ATP through the TCA cycle to prevent cell apoptosis and necrosis. Therefore, autophagy is more of an adaptive response which is beneficial to the cardiomyocytes during ischemia. However, whether the beneficial effects of AMPK are changed after the reperfusion and the regulatory role of AMPK in the reperfusion phase remains to be further studied.

### Other mTOR-Related Signaling Pathways

The non-coding RNAs not only regulate the upstream signaling molecules of the mTOR, but also regulate autophagy by directly targeting the mTOR. For example, the expression of autophagy molecular markers including Atg5, Atg 12 and LC3 was observed to increase after overexpression of miR-99a, while miR-99a can directly target mTOR and down-regulate the mTOR/P70/S6K signaling pathway to improve MIRI. It is speculated that miR-99a may increase autophagy and inhibit apoptosis *via* inhibition of the mTOR signaling pathway, thus improving cardiac function and survival rate in mouse models of myocardial infarction. miRNA-99a directly targets mTOR, inhibiting cell apoptosis and increases autophagy through the mTOR/P70/S6K signaling pathway, and improving the cardiac function and survival rate in a mouse model of myocardial infarction (Li et al., 2014). In addition, some non-coding RNAs can also regulate autophagy by indirectly regulating the mTOR. For example, High Mobility Group box-1 protein (HMGB1) is an important regulator of the H/R-induced apoptosis and autophagy. It can upregulate autophagy by activating AMPK and inhibiting mTOR, thus promoting the survival of myocardial cells after myocardial infarction (Foglio et al., 2017). Su et al. (2019) found that HMGB1 and Rac1 were the targets of miR-142-3p. Targeting lncRNA TUG1 or up-regulating miR-142-3p can inhibit autophagy and improve MIRI, which may be related to indirect regulation of the mTOR signaling pathway by the HMGB1. Moreover, another study found that miR-221 not only released the inhibition of the mTORC1/p-4EBP4 pathway by targeting DNA Damage inducible Transcript4 (Ddit4), a known mTORC1 suppressor and directly inhibiting the formation of the autophagosome, but also directly inhibited the autophagosome degradation, protecting myocardium from the I/R injury by reducing the formation of the Tp53inp1/P62 complex (Chen et al., 2016).

As mentioned above, mTOR is an important hub for the initiation of autophagy and is regulated by a variety of signaling pathways, especially PI3K/AKT and AMPK signaling pathways, which can sense the nutritional or energy status of the cells. AMPK signaling mediates autophagy during myocardial ischemia. The mTOR and various molecules in the related pathways are regulated by the non-coding RNAs and are expected to be the targets of non-coding RNAs in the treatment of MIRI.

## REGULATION OF NON-CODING RNAS IN DIFFERENT PARTS OF AUTOPHAGY

### The Induction of Autophagy

The induction of autophagy requires Atg1 kinase complex in yeast cells (Kamada et al., 2000). In mammalian cells, Unc-51-like kinase 1/2 (ULK1/2) are the earliest discovered homologs of the Atg1, and the ULK1 forms ULK1 complex with Atg13 and FIP200 (the focal adhesion kinase family-interacting protein of 200 kD, partial homolog of Atg17). The ULK1 complex *in vivo* is a bridge connecting the upstream nutrient or energy receptors, mTORs, and AMPK with downstream autophagosome formation. Atg13 can also promote the activation of ULK1/2 under nutrient-rich conditions, and ULK1 and Atg13 are phosphorylated by mTORC1 to inhibit their activities. Upon mTOR inhibition by stavation or rapamycin treatment, mTORC1 dissociates from the ULK1 kinase complex, activates ULK1, and then phosphorylates Atg13, Beclin-1, VPS34, and other downstream substrates to induce autophagy (Hosokawa et al., 2009).

As a central part of autophagy induction, ULK1 is regulated by many non-coding RNAs. For instance, miR-20b-5p can target ULK1and boycott ULK1 complex phosphorylation to inhibit autophagy, protecting human umbilical vein endothelial cells (HUVECs)from the H/R injury (Zhen et al., 2020). The inhibition of miR-30a caused a significant reduction in the protein levels of the ULK1 and Beclin-1 in myocardial tissue of MIRI rats, reduced autophagy and inhibited apoptosis, thereby protecting rat cardiomyocytes from the I/R injury (Xu et al., 2019). It is speculated that miR-30a can directly or indirectly inhibit the activation of ULK1 complex, thus reducing the formation of autophagosome.

### The Nucleation of the Autophagosome

The nucleation is the first step in the formation of double-membrane autophagosome which requires the activation of the class III phosphatidylinositol 3-kinase (PtdIns3K) complex, composed of mAtg14 (Atg14 in yeast cells), Beclin-1, vacuolar protein sorting 34 (VPS34), and p150 in mammals (He and Levine, 2010). The PtdIns3K complex generates 3-phosphate-phosphatidylinositol (PtdIns3P) (Burman and Ktistakis, 2010), and recruits multiple autophagy proteins such as zinc finger, WIPI1, and WIPI2 (two orthologs of Atg18) for nucleation (Proikas-Cezanne et al., 2004; Polson et al., 2010).

Atg14, also known as Beclin1-associated autophagy-related key regulator (Barkor), phosphorylates Beclin-1 through the interaction of its coiled-coil domain with the Beclin-1 (Fogel et al., 2013). It can also target the Ptdlns3K complex to possibly form autophagosome and increase the activity of Vps34, which is very important for the autophagy nucleation stage (Matsunaga et al., 2010; Zhao et al., 2014). (Liu et al., 2017) showed that miR-130a targeted ATG14, and the inhibition of the miR-130a increased the expression level of ATG14 and the phosphorylation level of Beclin-1 in H/R-induced primary cardiomyocytes, thus increasing autophagy and inhibiting myocardial cell apoptosis.

Beclin-1 is a mammalian homologous of Atg6/Vps30 in yeast, which is required for the autophagy nucleation stage (Erlich et al., 2007). Beclin-1 is highly expressed during reperfusion, so Beclin-1 mediated autophagy is particularly important in reperfusion (Matsui et al., 2007). Beclin-1 has been shown to interact with a protein called Rubicon to prevent the fusion of the autophagosomes with lysosomes and aggravate cell injury (Zhong et al., 2009). Besides, Beclin-1 is negatively regulated by bcl-2 anti-apoptotic family proteins. There is a complex crosstalk between Beclin-1 mediated autophagy and apoptosis, which can cooperate to aggravate the myocardial cell death (Gordy and He, 2012). Moreover, oxidative stress and ROS expression increased during reperfusion, further promoting Beclin-1 mediated autophagy and exacerbating MIRI. Perhaps, these molecular events are the reasons why autophagy causes damage than protection to cells in the reperfusion phase.

Many experimental results showed that downregulation of Beclin-1 inhibited autophagy and alleviated MIRI. For example, it was found from the I/R model that miR-384 (Zhang et al., 2019a) and miR-30a (Li et al., 2016) protected the cardiomyocytes from the I/R injury by targeting Beclin-1 and inhibiting superabundant autophagy. Additionally, Wang et al. (2020) investigated the effect of post-hypoxic adaptation (HPostC) on the H/R injury in matured cardiomyocytes in the d-galactose-induced cardiomyocyte aging model. The results showed that HPostC upregulated the transcription of miR-30a through DNA (cytosine-5)-methyltransferase 3B (DNMT3b)-mediated DNA hypomethylation of miR-30a promoter, thereby inhibiting BECN1(which encoding Beclin-1)-mediated autophagy and protecting the senescent cardiomyocytes from the H/R injury. Long non-coding RNAs, MALAT1(Wang S. et al., 2019), PVT1 (Ouyang et al., 2020), and AK088388 (Wang J. J. et al., 2019) promoted beclin-1 expression, upregulated autophagy, and increased the myocardial cell injury through competitive binding to miR-20b, miR-186, and miR-30a, respectively. In addition to the competitively binding with the miRNAs to regulate the expression of autophagy-related genes as ceRNAs, the lncRNA can also directly bind with the transcription factors to regulate autophagy. It was found that the lncRNA CAIF directly targeted the transcription factor p53 and blocked the combination of P53 with the cardiac protein promoter region after binding p53. Besides, P53 also modulate the transcription and activation of the myocardin, and the overexpression of myocardin markedly increased the expression of the Beclin-1, promoted the autophagy of cardiomyocytes, and aggravated the myocardial I/R injury (Liu et al., 2018). Notably, overexpression of myocardin did not affect the expression of Atg5, Atg7, Atg10, and Atg12 associated with autophagosome elongation. Therefore, it is speculated that the regulation of CAIF on P53 mainly affects the nucleation stage of autophagosome. Overexpression of CAIF alleviated H_2_O_2_-induced autophagic cell death through p53-myocardin axis.

However, in contrast, a study of ischemic injury found that enhanced Beclin-1-mediated autophagy reduced ischemic injury (Wang et al., 2014). In addition, Liu et al. (2019) found that miR-30a upregulation inhibited autophagy in the rat I/R model and H/R treated rat cardiomyocytes, which was contrary to the above conclusions. The downregulation of miR-30a increased the expression of Beclin-1 but enhanced the protective effect of autophagy on myocardia.

The above contradictory results may be attributed to the dual roles of Beclin-1 in promoting autophagosome formation and blocking autophagic flux. The role of Beclin-1 may be more complicated than expected, and its influence on autophagy needs to be further studied. Furthermore, whether the non-coding RNA affects MIRI through other pathways besides autophagy also requires more in-depth research.

### The Extension of Autophagosome

After the nucleation of the vesicles, the membrane elongation and enlargement of the autophagosomes are promoted by two ubiquitylation-like conjugation systems, Atg12-Atg5-Atg16L1 and Atg8/LC3-PE (Phosphatidylethanolamine).

#### Atg12-Atg5-Atg16L1 System

In the Atg12-Atg5-Atg16L1 system, Atg12 is first activated by Atg7 (E1-like enzyme), then transferred by Atg10 (E2-like enzyme) to bind with the Atg5, and then with Atg16L1 to generate the Atg12-Atg5-Atg16L1 complex, which is localized on the isolation membrane (also termed phagophore), promoting the expansion and extension of the autophagosome (He and Klionsky, 2009).

In this system, the non-coding RNAs regulate the recruitment of multiple Atg proteins to phagophores to participate in the formation of autophagosomes and regulate MIRI. For example, it was found that the miR-103a-3p (Zhang et al., 2019b), lncRNA-Galont (Yin et al., 2018), lncRNA FOXD3-AS1 (Tong et al., 2019) promoted autophagy by directly targeting Atg5 or indirectly regulating Atg5 expression as an endogenous sponge of miRNA, which aggravates MIRI.

Diabetes is a high-risk factor for adverse outcomes, with a higher incidence of ischemic heart disease-related morbidity and poorer prognosis in patients with diabetes compared to those without diabetes. The expression levels of lncRNA AK139328 and lncRNA NEAT1 were increased after the I/R treatment in the diabetic mice and rats, respectively (Ma et al., 2018; Yu et al., 2018). LncRNA AK139328 and lncRNA NEAT1, which were targeted miR-204-3p and Foxo1, respectively, enhanced autophagy by regulating Atg5 and Atg7 levels, which increased myocardial infarction size and aggravation of MIRI. Moreover, another study found that Neat1 was highly expressed in mice cardiomyocytes with ischemic cardiomyopathy/myocardial infarction, regulating the autophagy level through the control of Atg12 expression and related autophagy factors *via* targeting miR-378a-3p, which affected the proliferation, invasion, and metastasis of myocardial cells and alleviated hypoxia-induced myocardial cell injury (Zhao et al., 2020).

Atg7, the only E1-like enzyme in the autophagy system, can activate LC3 and Atg12 simultaneously, playing a core role in the two ubiquitination systems and promoting the extension of the autophagy membrane (Tanida et al., 1999). Studies have shown that lncRNA APF (Wang et al., 2015a) and circHIPK3 (Qiu et al., 2021) acted as functional sponges of the miR-188-3p and miR-20b-5p, respectively, and specifically regulated the expression of ATG7, inhibiting autophagy and apoptosis and further mitigating I/R injury. In contrast, Lv et al. (2021) showed that the upregulation of lncRNA H19 indirectly enhanced the expression of ATG7 by absorbing miR-143 like ceRNA, thus promoting autophagy and alleviating myocardial I/R injury. In addition, Ham et al. (2015) found that the autophagy pathway was activated, as evidenced by the increased expression of LC3A/B after treating cells with H_2_O_2_, while autophagy-related genes Atg5, Atg7, and Atg12 were dramatically downregulated in let-7b transfected human bone marrow mesenchymal stem cells (MSCs) under ROS enriched conditions, inhibiting the autophagy activity and attenuating I/R injury. Let-7b may inhibit autophagy by regulating Atg12-Atg5-Atg16L1 system.

Therefore, targeting proteins associated with this system can control MIRI, but more evidence is required to confirm whether it plays a protective or damaging role. Perhaps, controlling autophagy within a reasonable range by regulating the expression of miR-204 can alleviate MIRI.

#### Atg8(Lc3)-PE System

In the Atg8(LC3)-PE system, the LC3 protein is processed by cysteine protease Atg4 to produce cytosolic soluble LC3-I (Ichimura et al., 2000), and then covalently ligated with the phosphatidylethanolamine (PE) to form fat-soluble LC3-PE(LC3-II) under the action of Atg7 (E1-like enzyme) and Atg3 (E2-like enzyme) (Zhao et al., 2020), participating in the extension of the membrane (Harris and Rubinsztein, 2011). Usually, the ratio ofLC3- II to LC3-I can evaluate the degree of autophagy activation (Kroemer and Levine, 2008).

It was found that the downregulation of miR-204 (Xiao et al., 2011) increased the expression of LC3-II and enhanced autophagy, which aggravated the myocardial cell injury in the I/R model but did not affect the expression of LC3-I. Similarly, miR-214 regulated the ratio of LC3II/I, thus inhibiting autophagy and diminishing cell necrosis and I/R injury (Ghaderi et al., 2018).

Atg3 is an E2 enzyme involved in LC3 lipidation, which plays an important role in autophagy (Radoshevich et al., 2010). While P62 is expressed in a variety of cells and tissues and can bind to LC3 and ubiquitinated substrates, which are subsequently integrated into the autophagosomes and degraded (Pankiv et al., 2007). As such, the expression of P62 in cells is negatively correlated with autophagic activity and is often used as a marker for autophagy (Yoshii and Mizushima, 2017). It was found that in the H/R-treated human cardiomyocytes, the miR-431 expression was down-regulated, and autophagy was increased, as evidenced by the increased expression of ATG3 and LC3II/LC3I ratio, while p62 expression was down-regulated, contributing to the reduced survival rate of cardiomyocytes. ATG3 was found to be the target of miR-431. The overexpression of miR-431 at least partially alleviated the H/R-induced myocardial injury by regulating the expression ofATG3 (Zhou et al., 2021). Kong and colleagues found that the upregulation of lncRNA RMRP exacerbated MIRI by targetingATG3 through sponge-mediated miR-206 to promote autophagy (Kong et al., 2019).

In contrast to the above results, several studies are reporting that promoting autophagy alleviates MIRI in the Atg8(Lc3)-PE system. For example, autophagy was inhibited by the overexpression ofmiR-204, as evidenced by the repressions of the transformation from LC3-I to LC3-II and the expression of SIRT1 (Lee et al., 2008), an important regulator of autophagy, which yet had a protective effect on MIRI (Qiu et al., 2018). Similar to this result, the inhibition of miR-130a increased the ratio of LC3II/LC3I and reduced the expression level of P62, thereby promoting autophagy and enhancing cell viability (Liu et al., 2017). Also, it was found that the inhibition of miRNA-497 improved the cardiomyocyte I/R injury by inhibiting apoptosis and enhancing autophagy (Li et al., 2015). ATG4B, one of the homologs of ATG4, is an important autophagic enzyme in mammals (Yang et al., 2015), the downregulation of ATG4B inhibited rapamycin-induced autophagy (Wu et al., 2017). While the inhibition of miR-490-3p promoted the expression of LC3II, promoted autophagy, inhibited apoptosis through upregulation of ATG4B, thus reducing the infarct size and ameliorating MIRI (Wu et al., 2021).

The above-mentioned contradictory results may stem from different severity and the duration of ischemia/hypoxia, different predisposing factors (hypoxia, nutritional deficiency, infection, and reactive oxygen stress), different cell and animal models used, different use and formulation of the ischemic buffer, and different culture protocols. Hence, the various experimental backgrounds are likely to produce contradictory results.

### Fusion of Autophagosome and Lysosome

After the autophagosome matures, the outer membrane of the bilayer membrane and the lysosomal membrane fuse into one, forming an autophagolysosome with a monolayer membrane, degrading the phagocytosed intracellular materials and the inner autophagosome membrane. The specific mechanism underlying autophagosome-lysosome fusion is still unclear, but it is known that the proteins such as lysosomal membrane protein LAMP1/2, a microtubule scaffold protein, Rab, soluble N-ethylmaleimide-sensitive factor attachment protein receptor (SNARE), and ultraviolet radiation resistance-associated gene protein (UVRAG) are involved in the fusion process. For example, LAMP1/2 maintains an acidic environment in the lysosome, which is necessary for enzyme activity and hydrolysis; the SNARE increases the permeability of the membrane and creates membrane openings for the fusion of adjacent organelle contents. The STX17 is a type of SNARE called syntaxin 17, which is located on the mature outer membrane of the autophagosome and binds with the intracytoplasmic proteins SNAP29 and VAMP8, jointly mediating the fusion of the lysosomes and autophagosomes (Itakura et al., 2012). Rab protein is a small GTP binding protein that promotes the transport of the vesicles to target sites and anchor points (Stenmark and Olkkonen, 2001). UVRAG, an autophagy-promoting protein, binds with PtdIns3K complex to enhance the activity of Rab7 and promotes the fusion of autophagosomes and lysosomes (Jager et al., 2004; Liang et al., 2008).

Although no non-coding RNAs are directly involved in the fusion of autophagosome and lysosome, they have been found in MIRI. MiR-320a (Okato et al., 2016), miR-33 (Ouimet et al., 2016), miR-100-3p, and miR-16b (Ji et al., 2020), and LINC00511 (Peng et al., 2021) may have a negative regulatory effect on autophagy through the inhibition of LAMP1/2, V-SNARE protein, and vesicle-associated membrane protein 7 (VAMP7). For example, one of the direct targets of miR-33 is LAMP1, and the decreased expression of the LAMP1 regulated by miR-33 shows an inhibitory effect on autophagy. A previous study on tumors revealed that the circular RNA, circ_0000034 upregulated the STX17 level by repression of miR-361-3p (Jiang et al., 2021). LncRNA Xist upregulated the expression of STX17 *via* inhibiting miR-23b-3p/miR-29a-3p (Liu H. et al., 2020). Moreover, miR-520a-3p (Qi et al., 2020), miR-216b (Luo et al., 2018), and miR-125a (Kim et al., 2015) regulated autophagy by targeting UVRAG. Therefore, it is speculated that these non-coding RNAs play an important role in promoting the fusion of autophagosomes and lysosomes.

## Mitophagy

The above-mentioned autophagy pathways can degrade the components in the cytoplasm non-selectively, but selective autophagy is also involved in the degradation of the organelles and proteins. Selective autophagy mainly includes endoplasmic reticulum autophagy (Reticulophagy), mitochondrial autophagy (Mitophagy), and peroxisome autophagy (Pexophagy) and other autophagy (Li W. et al., 2021). The cardiomyocytes are rich in mitochondria, which are the main organelles that produce ROS. Mitophagy can maintain cell redox stability and reduce the cardiomyocyte death during the I/R by clearing the damaged mitochondria (Chun et al., 2016), which is critical in the pathogenesis of MIRI.

The non-coding RNAs play an important role in MIRI *via* the regulation of mitophagy through multiple autophagy regulators or transcription factors. For example, in the H/R-induced human adult cardiomyocytes (HACMs), miR-410 expression was declined, mitophagy was decreased, and the HMGB1 expression was downregulated, resulting in decreased cell viability. HMGB1 was found to be the targetof miR-410, and miR-410 may regulate HSPB1 activity, inhibit mitophagy, and aggravate MIRI by directly targeting HMGB1 (Yang et al., 2018).

Transcription factor FOXO3 induces the transcription of multiple autophagy genes including BNIP3, ATG12, LC3B, ATG4B, Beclin-1, vps34, and ULK2, and is also a pivotal regulator of the mitochondrial function (Song et al., 2017). Studies have found that the inhibition of miR-302a-3p targeting to the FOXO3, promoted mitophagy during MIRI, reduced mitochondrial dysfunction and I/R injury, and simultaneously maintains the integrity and purity of the mitochondria (Lv et al., 2020).

Further, a large number of studies demonstrated that PTEN-induced putative kinase 1 (Pink1) was essential in mitophagy and in scavenging damaged mitochondria through autophagy (Dagda et al., 2009; Michiorri et al., 2010; Gomez-Sanchez et al., 2016; Nguyen et al., 2016; Liu et al., 2021). For example, Pink1 promoted mitophagy and eliminated damaged mitochondria by promoting Parkin recruitment to the mitochondrial outer membrane and mitochondrial protein ubiquitination (Rakovic et al., 2013). Pink1 can also mediate the inhibition of mitophagy. Studies have shown that PINK_152_, the fission product of Pink1, inhibited the translocation of Parkin to the mitochondria, thus inhibiting the mitochondrial autophagy (Fedorowicz et al., 2014). Zhou and his colleagues found that Pink1 acted as a downstream target of the circRNA ACR to mediate autophagy and myocardial infarction. ACR regulated the expression of Pink1, inhibited cardiomyocytes and mitochondria autophagy, and reduced cell death by directly binding to DNA methyltransferase 3B (Dnmt3B), affecting Dnmt3B-mediated DNA methylation of Pink1. Besides, the study also found that the protein family with sequence similarity 65 member B (FAM65B) was a downstream target of Pink1, and FAM65B was phosphorylated at serine 46 by Pink1, inhibiting cardiac autophagy and cell death. Noteworthily, this study confirmed that circRNA regulated the gene expression by influencing the DNA methylation for the first time and revealed a new mechanism of the action of Pink1 (Zhou et al., 2019). This experiment verified that circRNA inhibited mitochondrial autophagy and alleviated MIRI by regulatingPink1 expression. Similarly, Zhang et al. showed that circPAN3/miR-421 signaling inhibited autophagy and improved MIRI by regulating the Pink1 (Zhang et al., 2021). Besides, Wang et al. found that miR-421 activated by E2F transcription factor 1 (E2F1) promoted mitochondrial fragmentation, apoptosis, and myocardial infarction through inhibition of the translation of Pink1 (Wang et al., 2015b).

To date, the mechanism underlying Pink1 in inhibiting or activating autophagy/mitophagy has not been fully understood, and more studies are needed to clarify it. At the same time, we also noted that similar to autophagy, mitophagy also plays a dual role in MIRI, which is speculated to be related to the stage of mitochondrial autophagy (ischemia or reperfusion), the degree of mitochondrial autophagy, the cell environment, and different stimuli.

## Conclusion and Prospects

**FIGURE 1:**
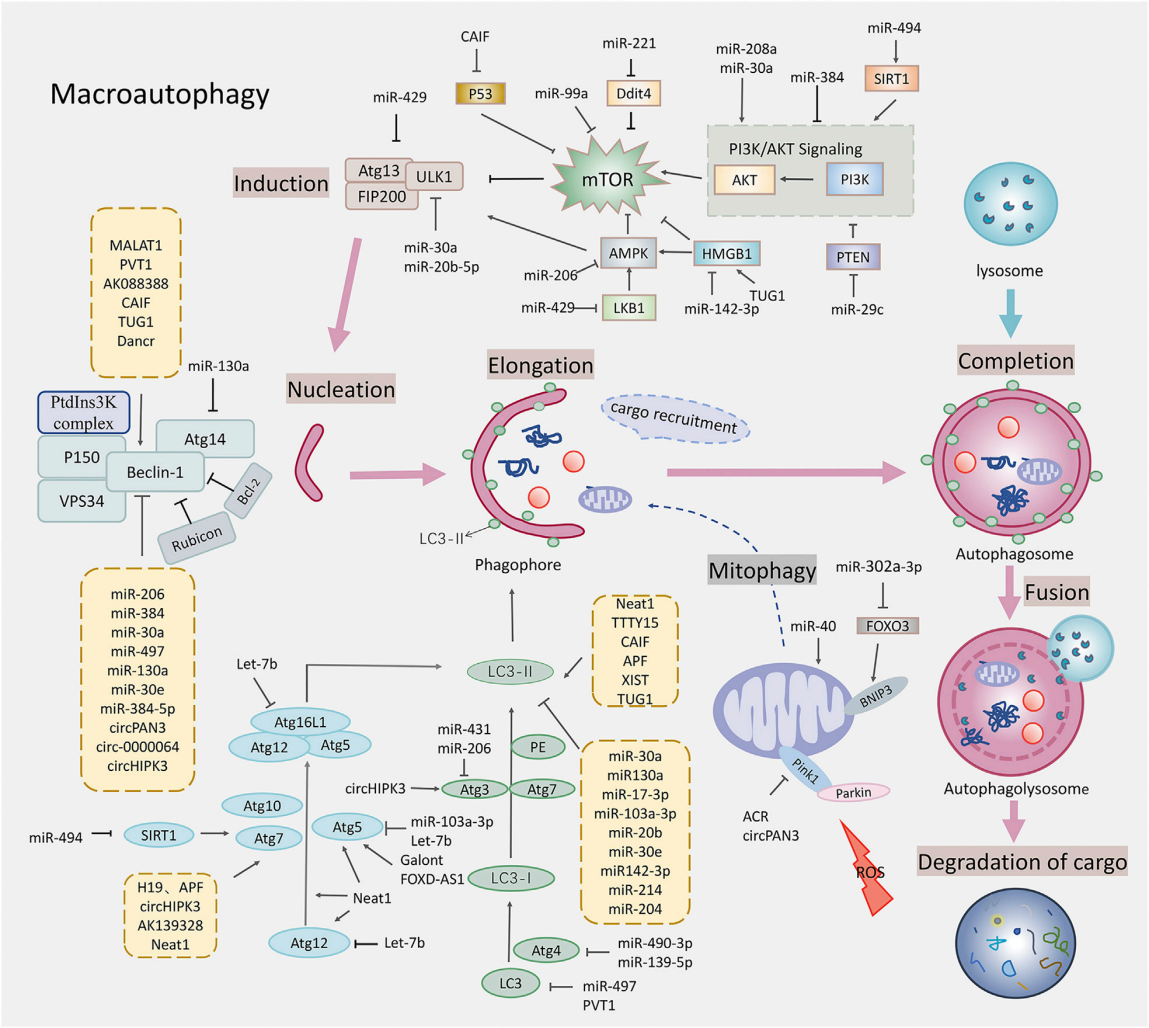
Schematic diagram of autophagy regulated by the non-coding RNAs in the myocardial ischemia-reperfusion injury. This figure shows that the non-coding RNAs affected the various stages of autophagy, regulating the targets related to the macroautophagy including the mTOR related signaling pathways, the ULK1 complex that is closely related to the Beclin-1, the vesicle nucleation promoted by the PtdIns3K complex, the autophagosome extension promoted by two ubiquitylation-like conjugation systems, the fusion of the autophagosomes with lysosomes, and mitophagy. Specific gene targets include the AMPK, mTOR, ULK1, Beclin-1, Atg5, Atg7, Atg3, Atg12, LC3, and Pink1.

In summary, non-coding RNAs affect the MIRI by regulating the mTOR-related signaling pathways, various links of autophagy, and mitochondrial autophagy in non-selective autophagy (see Figure 1). Targeting non-coding RNAs may become a novel therapeutic strategy for MIRI. Clinically, many non-coding RNAs are abnormally expressed in MIRI and other coronary heart diseases. For example, lncRNA NEAT (Zhao et al., 2020), miR-130a (Jansen et al., 2014), miR-30a (Shen et al., 2015), lncRNA MALAT1 (Vausort et al., 2014), and mitochondrial lncRNA LIPCAR (Li M. et al., 2018) were elevated in the peripheral blood of patients with coronary heart disease such as AMI and MIRI. MiR-497 (Voellenkle et al., 2010), miRNA-30e (Zheng et al., 2018), and lncRNA-ANRIL (Liu Z. F. et al., 2020) were significantly reduced in the serum of patients with coronary heart disease such as ischemic cardiomyopathy and MIRI. In animal experiments, a variety of drugs, including rosuvastatin (Wang X. et al., 2019), citrate (Xiang et al., 2020), puerarin (Han et al., 2021), morphine (Chen et al., 2018) and salidroside (Jin et al., 2021) etc., also ameliorated MIRI by regulating the expression of non-coding RNAs and biological processes such as autophagy. In addition, mesenchymal stem cells have the therapeutic potential to repair the myocardial injury while the microRNA-modified MSCs are more conducive and effective to repair the infarction-related injury and improve the cardiac function by elongating the survival of the transplanted cells and promoting the differentiation of myocardial cells (Boyle et al., 2010). Research on exosomes and adenoviruses as miRNA delivery vectors has also made further progress in assisting miRNA to treat MIRI. We summarize the non-coding RNAs that are potential drug targets in MIRI (see Table 1).

**TABLE 1:** Therapies for treating MIRI by targeting non-coding RNAs to regulate autophagy.

Treatment	Non-coding RNAs served as druggable targets	Mechanism	References
Puerarin	LncRNA ANRIL	Puerarin improved cardiomyocyte activity, and alleviated the MIRI by upregulation of ANRIL, which reduced LDH and MDA levels, inhibited the H/R induced autophagy and apoptosis of cardiomyocytes	Han et al. (2021)
Morphine	UCA1/miR-128	Morphine Postconditioning educed the size of myocardial infarction by inhibiting autophagy and apoptosis *via* regulation of UCA1/miR-128/HSP7 axis	Chen et al. (2018)
Citrate	miR-142-3p	Citrate pretreatment inhibited cell apoptosis and autophagy induced by H/R injury, decreased LDH and MDA levels, and alleviated H/R injury by miR-142-3p/Rac1 aix	Xiang et al. (2020)
Exosome	miR-30a	The miR-30a inhibitors carried by exosomes could reduce the contents of AST, CPK and MDA in serum, increase the activity of SOD, inhibit the expression of ULK1 and Beclin-1, reduce autophagy, and inhibit the apoptosis of myocardial cells in MIRI rats	Xu et al. (2019)
Bone Marrow Mesenchymal Stem Cell Derived Exosomal (BMMSCs)	miR-29c	Exosomes derived from BMMSCs contained high concentrations of miR-29C, which attenuated MIRI by inhibiting excessive autophagy *via* PTEN/Akt/mTOR signaling pathway	Li et al. (2020a)
Epigallocatechin gallate (EGCG)	miR-384-5p	EGCG preconditioning increased cell viability and reduced the size of myocardial infarction through adjusting miR-384-5p targets Beclin-1 to attenuate the expression of the I/R-induced autophagy flux *via* PI3K/Akt pathway	Zhang et al. (2019a)
salvianolic acid B (Sal B)	miR-30a	Sal B regulated Beclin-1 through miR-30/PI3K/Akt pathway, which inhibited I/R-induced autophagy, improved cell viability, reduced LDH leakage and alleviated MIRI.	Li et al. (2016)
Mesenchymal stem cells (MSCs)	let-7b	MSCs modified with let-7b could improve the survival rate of transplanted cells, reduce infarct size and promote the repair of myocardial injury by reducing the expression of autophagy-related genes BECN1, ATG5, ATG7 and ATG12	Ham et al. (2015)
Rosuvastatin (RS)	miR-17-3p	RS could promote autophagy, reduce LDH leakage and improve cell viability by inhibiting miR-17-3p. It could also reduce apoptosis by cleaved caspase-3/Cyto C signaling pathway	Wang X. et al. (2019)
Sevoflurane	miR-206	Sevoflurane post-conditioning could inhibit autophagy, apoptosis, and myocardial fibrosis, promote free radical clearance, promote free radical clearance, and improve MIRI by inhibiting miR-206 to activate AMPK/Nampt signaling pathway	Li et al. (2020b)
Sevoflurane	miR-208a	Sevoflurane post-conditioning could enhance SOD activity, inhibit the expression of MDA and facilitate the activation of the PI3K/AKT pathway *via* inhibition of miR-208a, thereby alleviating MIRI.	Shi et al. (2020)
Propofol	miR-20b-5p	Propofol preconditioning reduced apoptosis, and alleviated H/R injury *via* upregulation of mib-20b-5p, which targeted ULK1 to inhibit autophagy	Zhen et al. (2020)
Hypoxia postconditioning (HPostC)	miR-30a	HPostC promoted the expression of miR-30a through DNMT3b, thereby inhibiting BECN1-mediated autophagy and alleviating H/R injury. It is speculated that Ischemic postconditioning (IPostC) can also reduce I/R injury of elderly cardiac myocytes by targeting miR-30a	Wang et al. (2020)
Osthole	miR-30a	Osthole could promote autophagy by down-regulating miR-30a to reduce collagen content in ischemic reperfusion myocardium, inhibit myocardial apoptosis combined with myocardial injury, and alleviate MIRI.	Liu et al. (2019)
Salidroside	circ-0000064	Salidroside inhibited autophagy and apoptosis of cells by upregulation of CIRC-0000064, which improved myocardial function, decreased SOD, MDA, CK-MB and LDH levels, inhibited oxidative stress, reduced myocardial infarction size, thus alleviating MIRI	Jin et al. (2021)

Therefore, as discussed, the non-coding RNA is expected to be a new biomarker for the diagnosis and prediction of MIRI and a new candidate to treat MIRI. At present, studies on lncRNA and circRNA are still in the early stage. LncRNAs are promising biomarkers due to its tissue-specificity and relatively stable secondary structure, and circRNAs have shown great attractions as miRNA sponges or gene regulators to treat diseases due to their stable ring structure. As for miRNA therapies, some clinical trials on miRNA mimics and miRNA inhibitors have entered phase II or III, and many anti-miRNA therapies have been developed, such as antisense oligonucleotides, clonucleotides, miRNA sponges, CRISPR/Cas9 genome editing, etc. (Wang et al., 2014; Toden et al., 2021).

However, there are still many issues that need to be solved in identifying new non-coding RNA biomarker and developing non-coding RNA therapies. For example, the quantity and quality of the non-coding RNA in the peripheral blood and whether it is superior to the common clinical protein biomarkers still need more multicenter clinical verification with large samples. More clinical trials are also required to confirm the efficacy of these drugs. In addition, how to deliver drugs to specific target organs or target cells to exert their full efficacy, how to reduce the immunogenicity barriers of RNA therapy, how to improve the specificity of non-coding RNA to target, how to avoid the side effects of non-coding RNA therapy, and how to improve safety, all of them are great challenges in clinical applications (Winkle et al., 2021).

In the meantime, we also see that there are still many deficiencies in the research on the role of non-coding RNA in regulating autophagy in MIRI. For instance, in most studies on MIRI, the detection of autophagosome abundance only focused on the upregulation of autophagy rather than the blocking of autophagy degradation. Therefore, it was difficult to determine the cause of the increase in autophagy flux. In addition, the dichotomous roles of autophagy in cell protection and cell death make autophagy so controversial in the I/R model. Therefore, there is a need to establish and standardize both the *in vivo* and *in vitro* experimental models that reflect the different degrees of ischemic stress to accurately define the role of autophagy in MIRI. Furthermore, hypoxia is only a component of ischemia in the cell model, so the ischemic conditions at the organ level cannot be reproduced by using hypoxia alone. Therefore, the data derived from the cellular models must be interpreted with caution. More importantly, the experimental data obtained from the animal models have great limitations, whether the above-mentioned non-coding RNAs are conserved among species and whether these conclusions can be extrapolated to humans requires further investigation.

In general, autophagy is protective in the ischemic phase, while excessive autophagy in the reperfusion phase is detrimental in MIRI, and non-coding RNA mediates the MIRI by regulating autophagy and other processes. We summarized the effects of miRNA, lncRNA, and circRNA on MIRI respectively (Shown in Supplementary Table S1; Tables 2, 3). With the continuous innovation and development of the non-coding RNA, its regulatory mechanism in MIRI is expected to be further elucidated. Despite great challenges in research about the non-coding RNA, autophagy, and MIRI currently, we still believe that both the identification of non-coding RNA as a biomarker for detecting AMI or MIRI and its development as a targeted therapy will arouse great interest in the near future. Meanwhile, the in-depth research on autophagy and the mechanism underlying MIRI will also provide more and more powerful evidence for the targeted autophagy regulation of MIRI.

**TABLE 2:** The regulatory role of lncRNA in autophagy in the myocardial ischemia-reperfusion injury.

LncRNA	Sources/Cells used	Expression level	Targeted genes	Autophagy related targets	Effects on autophagy	Related signaling pathways	Effects on MIRI	References
Neat 1	SD rats	Up	—	Foxo1, Atg7, Atg5	promotion	—	Upregulation of Neat1 aggravated the myocardial infarction size in diabetic myocardial tissue treated with I/R	Ma et al. (2018)
AK088388	HL-1	Up	miR-30a	Beclin-1, LC3	promotion	—	Down-regulation of AK088388 weakened the autophagy during reperfusion and inhibited the myocardial cell death	Wang J. J. et al. (2019)
Neat 1	C57BL/6 mice	Up	miR-378a-3p	Atg12	Promotion	—	NEAT1 alleviated hypoxia-induced cardiomyocyte injury via miR-378-3p/Atg7 axis	Zhao et al. (2020)
PVT1	AC16	Up	miR-186	Beclin-1	Promotion	—	Knocking down PVT1 alleviated MIRI by inhibiting excessive autophagy	Ouyang et al. (2020)
HRIM	SD rats, H9c2	Up	—	LC3	Promotion	—	Inhibition of HRIM alleviated H/R induced injury by reducing excessive autophagy	Huang et al. (2018)
TTTY15	C57BL6/J mice, H9c2, HL-1	Up	miR-374a-5p	FOXO1	Promotion	—	Down-regulation of TTTY15 alleviated MIRI by inhibiting autophagy	Chen et al. (2021)
CAIF	C57BL/6 mice	Down	P53	myocardin	inhibition	—	Up-regulation of CAIF reduced the myocardial infarction size in the I/R hearts by inhibiting autophagy death	Liu et al. (2018)
APF	C57BL/6 mice	Up	miR-188-3p	ATG7	promotion	—	Up-regulation of APF promoted autophagy and cell death and enlarged the area of myocardial infarction	Wang et al. (2015a)
FOXD3-AS1	H9c2	Up	—	ATG5, LC3II, Beclin1, p62	Promotion	NF-κ B/COX2/iNOS	FOXD3-AS1 aggravated the H9C2 cell injury treated with I/R by promoting autophagy	Tong et al. (2019)
ANRIL	H9c2, SD rats	Down	—	LC3, Beclin-1	Inhibition	—	Up-regulation of ANRIL alleviated MIRI by inhibiting myocyte autophagy	Han et al. (2021)
H19	HL-1, C57BL/6 mice	Down	miR-143	ATG7	Promotion	—	Up-regulation of H19 triggered autophagy and alleviated H/R induced myocardial cell injury	Lv et al. (2021)
Galont	Mice	Up	miR-338	ATG5	Promotion	—	Up-regulation of Galont regulated ATG5, promoted autophagy, and aggravated the myocardial cell A/R injury	Yin et al. (2018)
PMRP	H9c2	Up	miR-206	ATG3	Promotion	PI3K/AKT/mTOR	Up-regulation of RMRP aggravated MIRI by down-regulation of miR-206 and up-regulation of ATG3	Kong et al. (2019)
MALAT1	H9c2	Up	miR-20b	Beclin1	Promotion	—	Up-regulated MALAT1 enhanced the autophagy and promoted the OGD/R induced myocardial cell injury	Wang S. et al. (2019)
UCA1	H9c2	Down	miR-128	HSP70	Inhibition	—	Up-regulation of UCA1 reduced the myocardial autophagy and apoptosis induced by myocardial I/R injury	Chen et al. (2018)
XIST	H9c2	Up	miR-133a	LC3, SOCS2	Promotion	—	Inhibition of XIST improved MIRI by inhibiting autophagy and regulating SOCS2	Li et al. (2019)
AK139328	C57BL/KsJ db/+ mice	Up	miR-204-3p	Atg7, Atg5, LC3, p62	Promotion	—	Silencing AK139328 alleviated MIRI in diabetic mice	Yu et al. (2018)
Dancr	H9c2	Down	miR-6324	Beclin-1, LC3	Inhibition	IRE1α/Xbp1	Up-regulated Dancr promoted autophagy and protected cardiomyocytes from ER stress injury	Li J. et al. (2021)
TUG1	C57BL/6J wild-type mice, HACMs	Up	miR-142-3p	HMGB1, Rac1	Promotion	—	Targeting TUG1 ameliorated myocardial I/R injury	Su et al. (2019)

**TABLE 3:** The regulatory role of circRNA in autophagy in the myocardial ischemia-reperfusion injury.

CircRNA	Sources/Cells used	Expression level	Targeted miRNA	Autophagy related targets	Effects on autophagy	Related signaling pathways	Effects on MIRI	References
circHIPK3	C57BL/6J mice, NMVCs	Up	miR-20b-5p	ATG5	promotion	—	Up-regulation of circHIPK3 promoted autophagy and apoptosis, and aggravated myocardial cell injury	Qiu et al. (2021)
circ_0000064	Wistar rats	Down	—	P62, LC3, Beclin-1	inhibition	—	Up-regulation of circ_0000064 improved MIRI by inhibiting autophagy and apoptosis	Jin et al. (2021)
ACR	C57BL/6 mice	Up	—	Pink1	inhibition	Pink1/FAM65B	Overexpression of ACR alleviated MIRI by inhibiting autophagy and cell death	Zhou et al. (2019)
circPAN3	C57BJ/6L mice, HCMs, HEK293	Down	miR-421	Pink1	inhibition	—	Overexpression of circPAN3 inhibited autophagy and improved MIRI	Zhang et al. (2021)

## Supplementary Material

Supplementary Table S1 The regulatory role of miRNAs in autophagy in the myocardial ischemia-reperfusion injury.

## Glossary

AKT: protein kinase B
AMI: acute myocardial infarction
AMPK: AMP-activated protein kinase
BMMSCs: mouse bone marrow mesenchymal stem cells
CK-MB: cardiac lactate creatine kinase-MB
CRL-1772: mouse myoblast cell line C2C12
Ddit4: DNA Damage inducible Transcript4
ceRNA: competitive endogenous RNA
Dnmt3B: DNA methyltransferase 3B; E2F1:E2F transcription factor 1
DNMT3b: DNA (cytosine-5)-methyltransferase 3B
FAM65B: family with sequence similarity 65 member B
HACMs: Human adult cardiac myocytes
HCMs: human cardiac myocytes
HEK293: embryonic kidney 293 cells
HMSCs: Human bone marrow-derived mesenchymal stem cells
HMGB1: High Mobility Group box-1 protein
HSPB1: heat shock protein family B (small) member 1
HUVECs: human umbilical vein endothelial cells
LAMP1/2: lysosomal-associated membrane protein 1/2
LDH: lactate dehydrogenase
LKB1: liver kinase B1
MDA: malondialdehyde
MIRI: myocardial ischemia-reperfusion injury
MO25: mouse protein 25
MSCs: mesenchymal stem cells
mTOR: mammalian target of rapamycin
Nampt: Nicotinamide phosphoribosyltransferase
NMVCs: Neonatal mouse ventricular cardiomyocytes
NRC: Neonatal rat cardiomyocytes
NRCMs: neonatal rat cardiomyocytes
NRVMs: Neonatal rat ventricular myocytes
OGT: O-GlcNac transferase
p-4EBP1: Phosphorylated 4E-binding protein 1
Pink1: PTEN-induced putative kinase 1
PIP2: phosphatidylinositol (4,5)-bisphosphate
PIP3: phosphatidylinositol (3,4,5)-trisphosphate
PI3K: Phosphatidylinositol 3-kinase
PPCI: primary percutaneous coronary intervention
PTEN: Phosphatase and tensin homolog deleted on chromosome 10
PtdIns3K: class III phosphatidylinositol 3-kinase
PtdIns3P: 3-phosphate-phosphatidylinositol
Rac1: RAS-related C3 botulinus toxin substrate 1
SD: Sprague-Dawley
SNARE: soluble N-ethylmaleimide-sensitive factor attachment protein receptor
SNAP29: synaptosomal-associated protein 29
SIRT1: silencing information regulator 1
Stx17: Syntaxin 17
ULK1/2: Unc-51-like kinase 1/2
UVRAG: ultraviolet radiation resistance-associated gene protein
VAMP: vesicle-associated membrane protein
VPS34: vacuolar protein sorting 34
WIPI1/2: WD repeat domain, phosphoinositide interacting 1/2
