# Supplementary material for: The Regulatory Role of Non-coding RNA in Autophagy in Myocardial Ischemia-Reperfusion Injury
Source: Front Pharmacol. 2022 Mar 17;13:822669. doi: 10.3389/fphar.2022.822669 (PMC8970621; doi:10.3389/fphar.2022.822669)
Supplement: Supplementary file 1 [file Table1.DOCX]

**Table 1: The regulatory role of miRNAs in autophagy in the myocardial ischemia-reperfusion injury**

| miRNA | Sources/Cells used | Expression level | Autophagy-related targets | Effects on autophagy | Related signaling pathways | Effects on MIRI | Reference |
| --- | --- | --- | --- | --- | --- | --- | --- |
| let-7b | HMSCs  SD rats | Down | Atg5, Atg7, Atg12, Beclin-1 | inhibition | - | Up-regulation of let-7b improved the survival rate of HMSCs after the transplantation in I/R injured heart | (Ham et al., 2015) |
| miR-99a | C57BL/6 Mice | Down | mTOR | promotion | mTOR/P70/S6K | Up-regulation of miR-99a improved the cardiac function and myocardial infarction survival rate | (Li et al., 2014) |
| miR-497 | NRCs | Down | LC3B | inhibition | - | Inhibition of miR-497 alleviated the I/R injury in myocardium | (Li et al., 2015) |
| miR-30a | C57BL/6 J mice | Down | Beclin-1, LC3-II | inhibition | PI3K/Akt | Up-regulation of miR-30a protected the myocardium from I/R injury | (Li et al., 2016) |
| miR-29c | BMMSCs | Up | - | inhibition | PTEN/Akt/mTOR | Up-regulation of miR-29C reduced the myocardial infarction size and I/R induced excessive autophagy, and protects the heart from I/R injury | (Li et al., 2020a) |
| miR-130 a | Rat primary myocardial cells 、SD rats | Up | ATG14, p-Beclin-1, LC3, P62 | inhibition | - | Inhibition of miR-130a increased the autophagy, inhibited the apoptosis, and alleviates H /R induced injury | (Liu et al., 2017) |
| miR-494 | H9c2 | Down | SIRT1 | inhibition | PI3K/AKT/mTOR | Up-regulation of miR-494 alleviated the H/ R-induced cell injury | (Ning et al., 2020) |
| miR-24 | Wild-type (WT) (C57BL/6J background) and diabetic mice | Down | OGT, ATG4A | inhibition | - | Up-regulation of miR-24 reduced the size of myocardial infarction and improved the I/R survival rate | (Wang et al., 2018) |
| miR -17- 3p | H9c2 | Up | LC3II, LC3I | promotion | - | Up-regulation of miR-17-3p improved the myocardial cell viability and reduced the MIRI | (Wang et al., 2019c) |
| miR-30a | H9c2 | Down | BECN1 | inhibition | - | Overexpression of miR-30a alleviated the H/R injury of senile cardiomyocytes | (Wang et al., 2020) |
| miR-204 | SD rats | Down | LC3-II | inhibition | - | Up-regulation of miR-204 reduced the myocardial cell I/R injury | (Xiao et al., 2011) |
| miR-103a-3p | H9c2 | Down | Beclin-1、Atg5、LC3 | inhibition | - | Up-regulation of miR-103a-3p alleviated the H/R-induced cell death by inhibiting autophagy | (Zhang et al., 2019b) |
| miR-20b-5p | HUVECs | Down | ULK1 | inhibition | - | Up-regulation of miR-20b inhibited the myocardial ischemia | (Zhen et al., 2020) |
| miR-30e | H9c2 | Down | Lc3, p62, Beclin-1 | inhibition | Notch1/Hes1/Akt | Down-regulation of miR-30e inhibited the apoptosis and oxidative stress induced by the myocardial infarction/reperfusion | (Zheng et al., 2018) |
| miR-325 | Mouse cardiomyocyte、 ARC knockout mice 、E2F1 knockout mice | Up | ARC | promotion | - | Knockdown of miR-325 inhibited the autophagy and cell death, and alleviated MIRI | (Bo et al., 2014) |
| miR-208a | SD rats | Up | Beklin-1, P62 | promotion | PI3K/AKT | Inhibition of miR-208a inhibited the autophagy and apoptosis, improved the myocardial cell viability, and alleviated MIRI | (Shi et al., 2020) |
| miR-30a | SD rats | Up | ULK1, Beclin-1 | promotion | - | Inhibition of miR-30a alleviated the myocardial apoptosis in the MIRI rats | (Xu et al., 2019) |
| miR-384-5p | H9c2 | Down | Beclin-1 | inhibition | PI3K/Akt | Upregulation of miR-384 protected the cardiomyocytes from MIRI by inhibiting excessive autophagy | (Zhang et al., 2019a) |
| miR-206 | SD rats | Up | p62, Beclin-1 | promotion | AMPK/Nampt | Up-regulation of miR-206 promoted the autophagy, apoptosis, fibrosis, and myocardial infarction size, and aggravated MIRI | (Li et al., 2020b) |
| miR-142-3p | NRCs | Down | Rac1 | inhibition | - | Up-regulation of miR-142-3p alleviated the H/R injury of the cardiomyocytes | (Xiang et al., 2020) |
| miR-34a | NRCMs、SD rats | Down | TNFα | inhibition | - | miR-34a mitigated the MIRI by targeting the TNFα to inhibit autophagy | (Shao et al., 2018) |
| miR-431 | Human cardiomyocytes | Down | ATG3 | inhibition | - | Up-regulation of miR-431 alleviated the H/R-induced myocardial injury by regulating the ATG3 | (Zhou et al., 2021) |
| miR-221 | NRVMs、myoblast、H9c2 | Down | Ddit4, Tp53inp1 | inhibition | DDIT4/mTORC1、Tp53inp1/p62 | Up-regulation of miR-221 protected the myocardium from H/R injury | (Chen et al., 2016) |
| miR-204 | H9c2 | Down | SIRT1 | inhibition | - | Overexpression of miR-204 protected the H9C2 cells from H/R-induced damage | (Qiu et al., 2018) |
| miR-429 | C57BL/6 mice、 SD rat | Down | MO25 | inhibition | MO25/LKB1/AMPK | Inhibition of miR-429 reduced the size of myocardial infarction in the I/R mice and improved the myocardial cell A/R injury | (Zhu and Hu, 2019) |
| miR-302a-3p | C57BL/6 J mice | Up | FOXO3 | inhibition | - | Down-regulation of miR-302a-3p promoted the mitophagy and improved MIRI | (Lv et al., 2020) |
| miR-410 | C57BL/6J wild-type mice、HACMs | Up | HMGB1 | inhibition | - | Up-regulation of miR-410 inhibited the mitophagy after myocardial I/R injury | (Yang et al., 2018) |
| miR-214 | CRL-1772 | Down | LC3 | inhibition | PKB/AKT | miR-214 rescued the I/R injured myoblasts by inhibiting necrosis and autophagy | (Ghaderi et al., 2018) |
| miR-30a | Wistar rats、SD rats | Up | Beclin-1 | inhibition | - | Down-regulation of miR-30a enhanced the autophagy and alleviated MIRI | (Wang et al., 2014) |
| miR-139-5p | H9c2 | Down | ATG4D | inhibition | - | Up-regulation of miR-139-5p inhibited the autophagy and mitigated MIRI | (Wang et al., 2017) |
| miR-490-3p | HEK293、C57BL/6 mice | Down | ATG4B | inhibition | - | Inhibition of miR-490-3p promoted the autophagy and alleviated MIRI | (Wu et al., 2021) |
